# Supplementary material for: Improved AlphaFold modeling with implicit experimental information
Source: Nat Methods. 2022 Oct 20;19(11):1376–82. doi: 10.1038/s41592-022-01645-6 (PMC9636017; doi:10.1038/s41592-022-01645-6)
Supplement: Supplementary file 1 — Reporting Summary [file 41592_2022_1645_MOESM1_ESM.pdf]

Corresponding author(s): Terwilliger, Thomas C

Last updated by author(s): 8/12/2022

Reporting Summary

Nature Research wishes to improve the reproducibility of the work that we publish. This form provides structure for consistency and transparency in reporting. For further information on Nature Research policies, see our Editorial Policies and the Editorial Policy Checklist.

Statistics

For all statistical analyses, confirm that the following items are present in the figure legend, table legend, main text, or Methods section.

n/a	Confirmed	
☒	☐	The exact sample size (*n*) for each experimental group/condition, given as a discrete number and unit of measurement
☒	☐	A statement on whether measurements were taken from distinct samples or whether the same sample was measured repeatedly
☒	☐	The statistical test(s) used AND whether they are one- or two-sided<br>*Only common tests should be described solely by name; describe more complex techniques in the Methods section.*
☒	☐	A description of all covariates tested
☒	☐	A description of any assumptions or corrections, such as tests of normality and adjustment for multiple comparisons
☒	☐	A full description of the statistical parameters including central tendency (e.g. means) or other basic estimates (e.g. regression coefficient) AND variation (e.g. standard deviation) or associated estimates of uncertainty (e.g. confidence intervals)
☒	☐	For null hypothesis testing, the test statistic (e.g. *F*, *t*, *r*) with confidence intervals, effect sizes, degrees of freedom and *P* value noted<br>*Give P values as exact values whenever suitable.*
☒	☐	For Bayesian analysis, information on the choice of priors and Markov chain Monte Carlo settings
☒	☐	For hierarchical and complex designs, identification of the appropriate level for tests and full reporting of outcomes
☒	☐	Estimates of effect sizes (e.g. Cohen's *d*, Pearson's *r*), indicating how they were calculated

Our web collection on statistics for biologists contains articles on many of the points above.

Software and code

Policy information about availability of computer code

Data collection	No data collection software was used.
Data analysis	Phenix software. Software, with documentation, instructions, test data, tests, example datasets, and tutorials is available at www.phenix-online.org. Version No custom modifications were made for this study.<br>ChimeraX. Version 1.2.5 used for display of models and density maps.

For manuscripts utilizing custom algorithms or software that are central to the research but not yet described in published literature, software must be made available to editors and reviewers. We strongly encourage code deposition in a community repository (e.g. GitHub). See the Nature Research guidelines for submitting code & software for further information.

Data

Policy information about availability of data

All manuscripts must include a data availability statement. This statement should provide the following information, where applicable:

- Accession codes, unique identifiers, or web links for publicly available datasets
- A list of figures that have associated raw data
- A description of any restrictions on data availability

All data were taken from the Protein Data Bank and the EM Data Bank
PDB accession codes:7mby 7me0 7ev9 7ls5 7eda 7lci 7lvr 7c2k 7m7b 7n8i 7mjs 7l1k 7l6u 7ku7 7kzz 7lx5 7brm 7lsx 7lc6 7mlz 7msw 7rb9 7bxt 7m9c 7lv9 (all directly downloadable with links such as: https://files.rcsb.org/download/7mby.pdb
EMDB entries (maps): 23750 23786 31325 23502 31062 23274 23541 30275 23709 24237 23883 23110 23208 23035 23093 23566 30160 23508 23269 23914 23970 24400 30237 23723 23530 . All downloadable directly from the EMDB with links such as: https://ftp.ebi.ac.uk/pub/databases/emdb/structures/EMD-23750/

map/emd_23750.map.gz.

Field-specific reporting

Please select the one below that is the best fit for your research. If you are not sure, read the appropriate sections before making your selection.

☒ Life sciences ☐ Behavioural & social sciences ☐ Ecological, evolutionary & environmental sciences

For a reference copy of the document with all sections, see nature.com/documents/nr-reporting-summary-flat.pdf

Life sciences study design

All studies must disclose on these points even when the disclosure is negative.

Sample size

The 25 maps and corresponding models shown in Fig. 1 and 3 were chosen in Aug. 2021 in a way that was intended to yield relatively representative recent structures in the PDB. We selected the first protein chain in the 25 most recently-deposited unique cryo-EM structures at the time with resolution of 4.5 Å or better and containing between 100 and 1000 residues. For this purpose, we considered two structures to be duplicates if the first protein chains matched in sequence at a level of 99% identity or greater. We included one pair of similar structures in the 25 structures chosen (7mlz and 7lx5). These differ in residues at the ends of the chain and differ also in that the SARS Cov-2 spike protein (the chain analyzed) is bound to different antibodies in the two structures. The PDB and EMDB accession numbers for these 25 structures are listed in Extended Data Table I.

The analysis of multiple structures (25) was carried out to demonstrate that the methods apply relatively generally to macromolecular cryo-EM structures. The number of structures analyzed (25) was chosen to be large enough to test whether the methods are limited to one or a few specific structures. The structures were chosen without regard to the contents of the structures (except to remove duplicates) to sample a variety of molecules).

The choice of structure and map to test model creation using AlphaFold trained without similar sequences in the PDB was made by selecting the (one) structure in CASP-14 that was determined by cryo-EM, classified as a "hard" target in CASP-1426, and for which experimental data is available in the EMDB and PDB. This structure was PDB entry 7bgl20, EMDB entry 12183. We chose domain 3 of chain a in this structure as AlphaFold performed poorly on this target in CASP-14 (rank of 78) compared to most other targets (rank of 1 for all other 7bgl targets).

Data exclusions

None

Replication

Replication was not carried out as a direct repetition of our calculations would give the identical result.

Randomization

We did not carry out randomization because our sampling procedure (taking all the unique deposits within a time frame) provides an unbiased sampling.

Blinding

The work was not blinded. This is a limitation of the analysis. The software was developed at the same time as the analysis and used some of the samples in the analysis in development. Consequently, it is possible that even though the same code and parameters are used for all the work shown here there may be choices made in parameters that improved results for these cases but that might not improve them for a completely new set of structures.

Reporting for specific materials, systems and methods

We require information from authors about some types of materials, experimental systems and methods used in many studies. Here, indicate whether each material, system or method listed is relevant to your study. If you are not sure if a list item applies to your research, read the appropriate section before selecting a response.

Materials & experimental systems

n/a	Involved in the study
☒	☐ Antibodies
☒	☐ Eukaryotic cell lines
☒	☐ Palaeontology and archaeology
☒	☐ Animals and other organisms
☒	☐ Human research participants
☒	☐ Clinical data
☒	☐ Dual use research of concern

Methods

n/a	Involved in the study
☒	☐ ChIP-seq
☒	☐ Flow cytometry
☒	☐ MRI-based neuroimaging
